# MILNP: Plant lncRNA–miRNA Interaction Prediction Based on Improved Linear Neighborhood Similarity and Label Propagation

**DOI:** 10.3389/fpls.2022.861886

**Published:** 2022-03-25

**Authors:** Lijun Cai, Mingyu Gao, Xuanbai Ren, Xiangzheng Fu, Junlin Xu, Peng Wang, Yifan Chen

**Affiliations:** College of Computer Science and Electronic Engineering, Hunan University, Changsha, China

**Keywords:** plant lncRNA-miRNA interaction, theoretical derivation, multilevel similarity, linear reconstruction, label propagation

## Abstract

Knowledge of the interactions between long non-coding RNAs (lncRNAs) and microRNAs (miRNAs) is the basis of understanding various biological activities and designing new drugs. Previous computational methods for predicting lncRNA–miRNA interactions lacked for plants, and they suffer from various limitations that affect the prediction accuracy and their applicability. Research on plant lncRNA–miRNA interactions is still in its infancy. In this paper, we propose an accurate predictor, MILNP, for predicting plant lncRNA–miRNA interactions based on improved linear neighborhood similarity measurement and linear neighborhood propagation algorithm. Specifically, we propose a novel similarity measure based on linear neighborhood similarity from multiple similarity profiles of lncRNAs and miRNAs and derive more precise neighborhood ranges so as to escape the limits of the existing methods. We then simultaneously update the lncRNA–miRNA interactions predicted from both similarity matrices based on label propagation. We comprehensively evaluate MILNP on the latest plant lncRNA-miRNA interaction benchmark datasets. The results demonstrate the superior performance of MILNP than the most up-to-date methods. What’s more, MILNP can be leveraged for isolated plant lncRNAs (or miRNAs). Case studies suggest that MILNP can identify novel plant lncRNA–miRNA interactions, which are confirmed by classical tools. The implementation is available on https://github.com/HerSwain/gra/tree/MILNP.

## Introduction

An increasing number of studies have shown that non-coding RNAs (ncRNAs), especially long non-coding RNAs (lncRNAs) and microRNAs (miRNA), act in various biological processes (Amin et al., 2019). miRNAs with a sequence length of approximately 22 nucleotides control post-transcriptional gene expression (DeVeale et al., 2021). lncRNAs, usually with a sequence length greater than 200 nucleotides, are widely engaged in essential regulatory processes (Ard et al., 2014; Chen et al., 2017; Fang et al., 2020; Statello et al., 2020; Goodall and Wickramasinghe, 2021). lncRNAs control the expression of miRNAs to influence the expression of their target genes: lncRNAs compete with mRNA for miRNAs, thereby regulating miRNA-mediated target inhibition (Geisler and Coller, 2013). For example, in the lumbar intervertebral disk degeneration (Zhu et al., 2019), lncRNAs may act as competing endogenous RNA (ceRNAs) that bind competitively to miRNAs through their miRNA response elements, thereby regulating the expression of miRNA-targeted mRNAs. miRNAs and lncRNAs interact with each other to exert higher levels of post-transcriptional regulation.

As computer technology advances rapidly, numerous methods are employed to study miRNAs, lncRNAs, and proteins, as well as their interactions (Fu et al., 2019, 2020; Cai et al., 2020a,b, 2021; Dai et al., 2021; Li P. et al., 2021; Liu et al., 2021; Rahaman et al., 2021; Song et al., 2021; Tan et al., 2021; Zhang C. L. et al., 2021; Zhang et al., 2022). With regard to miRNAs, a miRNA that is positively selected during human evolution is identified to regulate energy expenditure, and the relevance of this positively selected locus to metabolic disorders may explain the link between this locus and metabolic diseases (Stower, 2020). With regard to lncRNAs, a lncRNA GCMA activated by SP1 acts as a competing endogenous RNA in gastric cancer *via* competition for miR-124 and miR-34a to promote tumor metastasis (Tian et al., 2020). With regard to interactions between lncRNAs and proteins, lncRNA DIGIT regulates endoderm differentiation by promoting the formation of phase-separated condensates of bromodomain and the extraterminal domain protein BRD3 (Daneshvar et al., 2020). With regard to interactions between lncRNAs and miRNAs, the targeting lnc–MGC inhibits host lnc–MGC expression while suppressing the expression of key cluster miRNAs in the kidneys and preventing early diabetic nephropathy (Allison, 2016). Studies such as these are abundant and have made important contributions.

Although predictions about lncRNA–miRNA interactions exist, most of them are not about plants (Jiang et al., 2018, 2019; Ayachit et al., 2020; Banerjee et al., 2020; Ma et al., 2020; Qazi et al., 2020; Shen et al., 2020; Aglawe et al., 2021). The confirmed plant lncRNA–miRNA interactions are very limited and have been barely covered. For instance, the NPInter4.0 (Teng et al., 2020) documents extensive functional interactions between ncRNAs and molecules of over 30 species, yet only two of them are plants. From 71 RNA–RNA interactions for the two plants, only one of them is a miRNA–lncRNA interaction. It is no secret that the mechanisms of plant miRNA–lncRNA interactions remain elusive. Also, lncRNAs are characterized by low sequence conservation, especially among distantly related species.: The lncRNA molecules of different species or the same species may vary in terms of amino acid and nucleotide fragments during biological evolution, which entails that predictions obtained from animal studies are not guaranteed to be applicable in plants (Noviello et al., 2018). As a result, conclusions about the mechanism of plant lncRNA–miRNA interactions cannot be completely copied from animals and must be explored.

Studies on lncRNA–miRNA interactions generally fall under two categories, namely, bioinformatics-based machine learning methods and similarity network-based methods (Liu et al., 2017, 2020; Peng et al., 2017; Zeng et al., 2017, 2018, 2019; Zhao et al., 2020; Chen et al., 2021; Singh et al., 2021; Wang et al., 2021; Zhang Y. et al., 2021; Zhou et al., 2021; Zhu et al., 2021). The former extracts biological features and trains models to obtain dichotomous results (i.e., the output is whether lncRNA and miRNA interact) (Intell, 2019; Li J. et al., 2021). By comparison, the latter computes single or multiple correlation similarity matrices to obtain the final predictions (Wang et al., 2014). The works that use machine learning, even deep learning methods, do succeed. However, machine learning is flawed in terms of two aspects (Peng et al., 2018; Zhang et al., 2019b). First, it relies on data features. For certain lncRNAs or miRNAs, they may not have expression profiles or target genes. In this situation, machine-learning methods are not applicable. Moreover, for some isolated lncRNAs or miRNAs that do not have any interactions with miRNAs or lncRNAs at all, they have difficulty forecasting any unknown interaction. By contrast, similarity network-based approaches can address such imperfections. Constructing similarity networks does not necessarily depend on specific data features (Zhang et al., 2019b), and it is able to predict isolated lncRNAs and miRNAs solely on the basis of sequence information. Linear neighborhood similarity, which refers to selecting the most appropriate neighborhoods for linear reconstruction, as a new similarity measurement perspective, is currently gaining momentum in bioinformatics (Li et al., 2018; Zhang et al., 2018a,2019a; Xie et al., 2020; Zhang W. et al., 2021; Jia and Luan, 2022; Zhu et al., 2022), such as LPLNP (Zhang et al., 2018a) and MPLPLNP (Jia and Luan, 2022), in predicting lncRNA–protein interactions, and FLNSNLI (Zhang W. et al., 2021) and LPLNS (Li et al., 2018) in predicting miRNA–disease associations. To the best of our knowledge, no similarity network-based method is available to date for predicting lncRNA–miRNA interactions in plants. Owing to the imperfections of machine-learning methods and the necessity to independently detect plant lncRNA–miRNA interactions, novel and effective methods must be constructed.

In this study, we hypothesize that lncRNA–miRNA interactions with highly similar lncRNAs will have similar interaction or non-interaction patterns with miRNAs. Under this assumption, a multi-source information-based linear neighborhood propagation method (MILNP) is proposed. The similarity is calculated through our improved linear neighborhood similarity (ILNS) algorithm, where ILNS has the advantage of obtaining a more accurate neighborhood range over the pre-improvement. First, multidimensional features are separately extracted from the sequences of lncRNAs and miRNAs to calculate sequence similarity, whereas interaction profile similarity is obtained using their interactions. These two similarities are then combined to obtain integrated similarity. Label propagation based on the integrated similarity is used to calculate individually the prediction matrix of lncRNAs and the prediction matrix of miRNAs. Finally, the two prediction matrices are summed by taking different weights to obtain the final prediction. The contribution consists of the following components.

• We proposed a novel similarity measurement, ILNS, for calculating multiple similarity profiles.
• We constructed MILNP based on ILNS to predict lncRNA–miRNA interactions and discovered new interactions in the plant.
• High-accuracy prediction results in multiple experiments and showed superiority over the existing methods and reliability for finding new interactions of MILNP.

## Datasets and Methods

### Dataset Construction

The original data used herein are derived from a previous study (Kang et al., 2020) that investigated plant lncRNA–miRNA interactions. miRNA sequences are downloaded from miRBase22.1 (Kozomara et al., 2019), whereas lncRNA sequences are downloaded from GreeNC1.12 (Paytuvi-Gallart et al., 2019) and CANTATAdb2.0 (Szcześniak et al., 2019). Datasets of lncRNA–miRNA interaction from *Arabidopsis thaliana*, *Glycine max*, and *Medicago truncatula* are chosen with 2,500 positive samples from the positive dataset of each species, for a total of 7,500 positive samples. Similarly, 2,500 negative samples from the negative dataset of each species are chosen, also for a total of 7,500 negative samples. These positive and negative samples are intermixed as the training–validation set to avoid imbalance in sample distribution.

**FIGURE 1:**
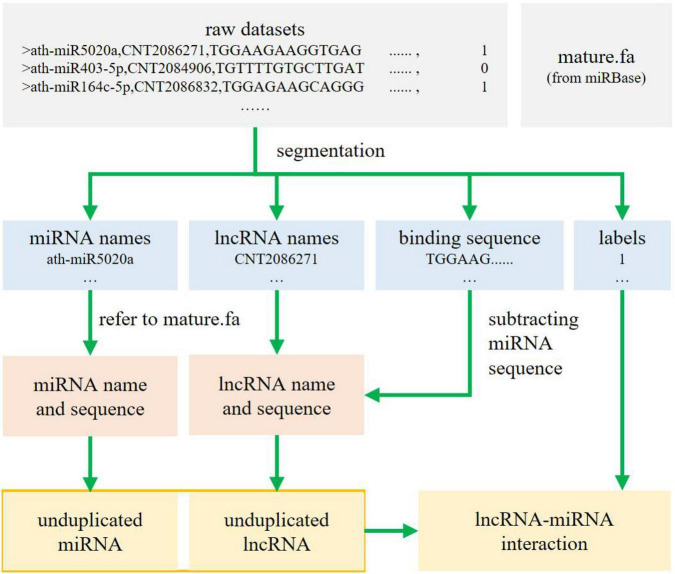
Dataset processing.

The dataset consists of five parts per sample: the symbol, the miRNA name, the lncRNA name, the sequence yielded from combining the miRNA sequence with the lncRNA sequence, and the sample label (0 for the absence of interaction, and 1 for the presence of an interaction). However, such a format is inappropriate for the method we applied herein. The processing is as follows. First, the original sequence binding files are separated by name, sequenced, and labeled to obtain the name of miRNA, name of lncRNA, binding sequences of miRNA and lncRNA, and labels. Second, all miRNA sequences are found according to the miRNA name order by checking against the reference documents from miRBase22.1 (Kozomara et al., 2019), and the lncRNA sequence of each line is intercepted according to the binding file, which happens to follow the lncRNA name order. Third, all miRNAs and lncRNAs (originally 15,000 lines each) are de-duplicated to obtain 1,340 unduplicated miRNAs and 7,963 unduplicated lncRNAs. Finally, the serial numbers of the remaining miRNAs and lncRNAs are determined, and the miRNA–lncRNA interaction matrix is drawn in accordance with the tag file. A rough workflow is shown in Figure 1.

Given that multiple pieces of information are required to calculate the interactions when we adopt the linear neighborhood similarity method, we also utilize these features that represent sequence information in addition to the interaction matrix. Owing to the existence of orphaned miRNAs and lncRNAs, the sequence is more versatile than the interaction. We collate the *k*-mer frequency (Ahmed et al., 2020), GC content, number of base pairs, and MFE (Negri et al., 2018) of miRNAs and lncRNAs according to the de-duplication files, the original files, and the features here.

The data are summarized in Table 1.

**TABLE 1:** Dataset composition.

Molecule	Quantity	Features
lncRNA	7,963	*k*-mer frequency (*k* = 1, *k* = 2, *k* = 3), GC content, number of base pairs, MFE
miRNA	1,340	*k*-mer frequency (*k* = 1, *k* = 2), GC content, number of base pairs, MFE
interaction	7,500	-

### Exact Linear Neighborhood Propagation Method Based on Combined Information

#### Improved Linear Neighborhood Similarity Measure

A matrix *M* is formed by *n*-dimensional feature vectors *x*_1_, …, *x*_*m*_ in space, where each row *x*_*i*_ (*x*_*i*1_, *x*_*i*2_, …, *x*_*in*_) is regarded as a data point, and we assume that we can gather the attribute parts of other data points to get the current one. Adjacent data points are usually viewed as possessing similar properties. Hence, the neighbors can be selected as the contributing force for the reconstruction of the point, whereas other irrelevant data points participate in the calculation but are assigned a weight of 0. For *x*_*i*_, the common calculation method for selecting neighbors is Euclidean distance. Considering that various features, such as GC content and MFE, characterize the components of the data point in these dimensions and that similar data points are assumed to eventually form multidimensional vectors with similar directions, the Cosine distance is then chosen to select neighbors *N*_1_(*x*_*i*_) (a total of *n*_1_), and then the Euclidean distance is chosen to select nearer neighbors *N*_2_(*x*_*i*_) (a total of *n*_2_) on the basis of the Cosine distance to select more exact neighbors. The latter is a subset of the former, ensuring that neighbors are nearer in both direction and position. The order of the two can be swapped since the final selected neighborhood is the same. The percentages of neighbors are expressed by *K*_1_ and *K*_2_ where *K*_1_ = *n*_1_/*m* and *K*_2_ = *n*_2_/*n*_1_.

To minimize the reconstruction error for *m* data points, we propose the objective function:


(1) $$\begin{array}{c} \min_{G,W}\frac{1}{2}{||2M-\left(C_{1}⊙G\right)M-\left(C_{2}⊙W\right)M||}_{F}^{2}\\ +\frac{\mu }{2}\sum_{i=1}^{m}{||\left(C_{1}⊙G\right)e+\left(C_{2}⊙W\right)e||}_{2}^{2}\\ s.t.\left(C_{1}⊙G\right)e=\left(C_{2}⊙W\right)e=e,G\geq 0,W\geq 0 \end{array}$$

where *M* is an *m* × *n* matrix in the feature space, and both *C*_1_ and *C*_2_ are indicator matrices that separately indicate whether they are neighbors on the basis of the Cosine distance and nearer neighbors on the basis of the Euclidean distance. *G* and *W* are both weight matrices. Here, μ is a weight parameter, and *e* is an *m* × 1 column vector with all elements being 1. ‖•‖_*F*_ is to obtain the Frobenius norm of a matrix. ‖•‖_2_ is to obtain the 2-norm of a vector. The first term of the objective function is to get the optimal weight matrix to minimize the reconstruction error of all data points, whereas the second term is to reduce overfitting during the reconstruction. For the weight matrices *G* and *W*, the elements are non-negative.

Given that the first neighbors are required to find the second ones, the objective function must be decomposed:


(2) $$\begin{array}{c} f\left(G\right)=\frac{1}{2}{||M-\left(C_{1}⊙G\right)M||}_{F}^{2}+\frac{\mu }{2}\sum_{i=1}^{m}{||\left(C_{1}⊙G\right)e||}_{2}^{2}\\ s.t.\left(C_{1}⊙G\right)e=e,G\geq 0 \end{array}$$

The Lagrange multiplier method is used to solve Equation (2), which then has the following form:


$$\begin{array}{c} \min_{G}f\left(G\right)\\ s.t.g\left(G\right)=e-\left(C_{1}⊙G\right)e=0\\ h\left(G\right)=-G\leq 0 \end{array}.$$

Then


(3) $$\begin{array}{c} L\left(G,\lambda_{1},\lambda_{2}\right)=f\left(G\right)+\lambda_{1}^{T}h\left(G\right)+\lambda_{2}^{T}g\left(G\right)\\ \;=\frac{1}{2}||X-\left(C_{1}⊙G\right)M|{|}_{F}^{2}+\frac{\mu }{2}\sum_{i=1}^{m}\left(C_{1}⊙G\right)e|{|}_{2}^{2}\\ \;+\lambda_{1}^{T}\left(-G\right)+\lambda_{2}^{T}\left(e-\left(C_{1}⊙G\right)e\right) \end{array}$$

where λ_1_ and λ_2_ are Lagrange factors.

According to the Karush–Kuhn–Tucker condition (Kjeldsen, 2000), the following conditions must be satisfied to determine the optimal value:


$$\left\{ \begin{array}{c} \nabla_{G}L=0\\ g\left(G\right)=0\\ \lambda_{1}^{T}h\left(G\right)=0 \end{array}\right..$$

The partial derivative of *L* is determined with respect to *G*:


(4) $$\begin{array}{c} L\left(G,\lambda_{1},\lambda_{2}\right)\\ =\frac{1}{2}{||M-\left(C_{1}⊙G\right)M||}_{F}^{2}+\frac{\mu }{2}\sum_{i=1}^{m}{||\left(C_{1}⊙G\right)e||}_{2}^{2}\\ +\lambda_{1}^{T}\left(-G\right)+\lambda_{2}^{T}\left(e-\left(C_{1}⊙G\right)e\right)\\ =\frac{1}{2}tr\left({\left(M-\left(C_{1}⊙G\right)M\right)}^{T}\left(M-\left(C_{1}⊙G\right)M\right)\right)\\ +\frac{\mu }{2}\sum_{i=1}^{m}{\left(\left(C_{1}⊙G\right)e\right)}^{T}\left(\left(C_{1}⊙G\right)e\right)-\lambda_{1}^{T}G-\lambda_{2}^{T}\left(\left(C_{1}⊙G\right)e-e\right)\\ =\frac{1}{2}[tr\left(M^{T}M\right)-tr\left(M^{T}\left(\left(C_{1}⊙G\right)M\right)\right)-tr\left({\left(\left(C_{1}⊙G\right)M\right)}^{T}M\right)\\ +tr\left({\left(\left(C_{1}⊙G\right)M\right)}^{T}\left(\left(C_{1}⊙G\right)M\right)\right)]\\ +\frac{\mu^{\prime }}{2}{\left(\left(C_{1}⊙G\right)e\right)}^{T}\left(\left(C_{1}⊙G\right)e\right)\\ -\lambda_{1}^{T}G-\lambda_{2}^{T}\left(\left(C_{1}⊙G\right)e-e\right) \end{array}$$

(5) $$\begin{array}{c} \nabla_{G}L\\ \;=\frac{1}{2}[\frac{\partial tr\left(M^{T}M\right)}{\partial G}-\frac{\partial tr\left(M^{T}\left(\left(C_{1}⊙G\right)M\right)\right)}{\partial G}\\ \;-\frac{\partial tr\left({\left(\left(C_{1}⊙G\right)M\right)}^{T}M\right)}{\partial G}\\ \;+\frac{\partial tr\left({\left(\left(C_{1}⊙G\right)M\right)}^{T}\left(\left(C_{1}⊙G\right)M\right)\right)}{\partial G}]\\ \;+\frac{\mu^{\prime }}{2}\left(\frac{\partial tr{\left(\left(C_{1}⊙G\right)e\right)}^{T}\left(\left(C_{1}⊙G\right)e\right)}{\partial G}\right)\\ \;-\frac{\partial tr\left(\lambda_{1}^{T}G\right)}{\partial G}-\frac{\partial tr\left(\lambda_{2}^{T}\left(\left(C_{1}⊙G\right)e-e\right)\right)}{\partial G}\\ \;=\frac{1}{2}[0-C_{1}⊙MM^{T}-\frac{\partial tr\left(M^{T}\left(\left(C_{1}⊙G\right)M\right)\right)}{\partial G}\\ \;+\frac{\partial tr\left(M^{T}{\left(C_{1}⊙G\right)}^{T}\left(C_{1}⊙G\right)M\right)}{\partial G}]\\ \;+\frac{\mu^{\prime }}{2}\left(\frac{\partial tr(e^{T}\left(C_{1}⊙G^{T}\left(C_{1}⊙G\right)e\right)}{\partial G}\right)\\ \;-\lambda_{1}-C_{1}⊙\left(\lambda_{2}e^{T}\right)\\ \;=\frac{1}{2}[-C_{1}⊙MM^{T}-C_{1}⊙MM^{T}\\ \;+2C_{1}⊙\left(C_{1}⊙G\right)MM^{T}]\\ \;+\frac{\mu^{\prime }}{2}\times 2C_{1}⊙\left(C_{1}⊙G\right)ee^{T}\\ \;-\lambda_{1}-C_{1}⊙\left(\lambda_{2}e^{t}\right)=C_{1}⊙\left(C_{1}⊙G\right)MM^{T}\\ \;-C_{1}⊙MM^{T}+\mu^{\prime }C_{1}⊙\left(C_{1}⊙G\right)ee^{T}\\ \;-\lambda -C_{1}⊙\left(\lambda_{2}e^{t}\right)\\ \;=C_{1}⊙(\left(C_{1}⊙G\right)MM^{T}+\mu^{\prime }\left(C_{1}⊙G\right)ee^{T}-MM^{T}\\ \;-\lambda_{2}e^{T})-\lambda {}_{1} \end{array}$$

Then


$$\left\{ \begin{array}{c} \nabla_{G}L=C_{1}⊙(\left(C_{1}⊙G\right)MM^{T}+\\ \mu^{\prime }\left(C_{1}⊙G\right)ee^{T}-MM^{T}-\lambda_{2}e^{T})-\lambda {}_{1}=0\\ e-\left(C_{1}⊙G\right)e=0\\ \lambda_{1}^{T}\left(-G\right)=0 \end{array}\right.$$

If λ*_1_*^T^** = 0, then there will be


(6) $$\begin{array}{c} \nabla_{G}L=C_{1}⊙(\left(C_{1}⊙G\right)MM^{T}\\ +\mu^{\prime }\left(C_{1}⊙G\right)ee^{T}-MM^{T}-\lambda_{2}e^{T})=0 \end{array}$$

In that case,


(7) $$G_{ij}=\frac{G_{ij}{\left(MM^{T}+\lambda_{2}e^{T}\right)}_{ij}}{{\left(\left(C_{1}⊙G\right)MM^{T}+\mu \left(C_{1}⊙G\right)ee^{T}\right)}_{ij}}$$

If λ*_1_*^T^** ≠ 0, then there will be *G* = 0. Thus, *G*_*ij*_ = 0.

Given the relevance of data point-based reconfiguration that *G*_*ij*_ ≠ 0 when *x*_*j*_∈*N*_1_(*x*_*i*_), the solution is


(8) $$G_{ij}=\left\{ \begin{array}{c} \frac{G_{ij}{\left(MM^{T}+\lambda_{2}e^{T}\right)}_{ij}}{{\left(\left(C_{1}⊙G\right)MM^{T}+\mu \left(C_{1}⊙G\right)ee^{T}\right)}_{ij}},x_{j}\in N_{1}\left(x_{i}\right)\\ 0\;,x_{j}\notin N_{1}\left(x_{i}\right) \end{array}\right.$$

Thus far, the iterative form with unknown parameters has been obtained. We inscribe its equivalent form for Equation (2) to obtain the following parameter:


(9) $$\begin{array}{c} \min_{G_{i}}L^{i}=\frac{1}{2}||x_{i}-\sum j:x_{j}\in N_{1}\left(x_{i}\right)G_{i,j}|{|}^{2}\\ +\frac{\mu }{2}{\left(\sum j:x_{j}\in N_{1}\left(x_{i}\right)|G_{i,j}|\right)}^{2}=\frac{1}{2}\vartheta_{i}^{T}Gr^{i}\vartheta_{i}+\frac{\mu }{2}||\vartheta_{i}|{|}_{1}^{2}\\ s.t.e^{T}\vartheta_{i}=1,\vartheta_{i}\geq 0 \end{array}$$

where *Gr^i^* is the gram matrix. If *x*_*j*_∈*N*_1_(*x*_*i*_) and *x*_*k*_∈*N*_1_(*x*_*i*_), then *Gr*_*j*_, *_*k*_* = (*x*_*i*_ – *x*_*j*_)*^T^*(*x*_*i*_ – *x*_*k*_). Otherwise, *Gr*_*j, k*_ = 0. Solving Equation (9) using the Lagrange multiplier method yields


(10) $$L^{i}=\frac{1}{2}\vartheta_{i}^{T}Gr^{i}\vartheta_{i}+\frac{\mu }{2}{||\vartheta_{i}||}_{1}^{2}-\lambda_{i}\left(e^{T}\vartheta_{i}-1\right)-\eta^{T}\vartheta_{i}$$

By taking the partial derivatives of *g* and λ, λ*_*i*_* can be obtained:


(11) $$\lambda_{i}=\left(\vartheta_{i}^{T}Gr^{i}\vartheta_{i}+\mu {\left(e^{T}\vartheta_{i}\right)}^{2}\right)/e^{T}\vartheta_{i}$$

where the reconstruction error is close to none, i.e., $1/2\vartheta_{i}^{T}Gr^{i}\vartheta_{i}\approx 0$. According to the Lagrange multiplier method, *e*^*T*^ϑ_*i*_−1 = 0. Thus, λ*_*i*_* = μ. If λ = μ × *e*, *G* can be represented as


(12) $$G_{ij}=\left\{ \begin{array}{c} \frac{G_{ij}{\left(MM^{T}+\mu ee^{T}\right)}_{ij}}{{\left(\left(C_{1}⊙G\right)MM^{T}+\mu \left(C_{1}⊙G\right)ee^{T}\right)}_{ij}}\;,x_{j}\in N_{1}\left(x_{i}\right)\\ 0\;,x_{j}\notin N_{1}\left(x_{i}\right) \end{array}\right.$$

The similarity matrix based on the Cosine distance is then acquired through iteration until convergence. Similarly, *W* is obtained with respect to *G*:


(13) $$W_{ij}=\left\{ \begin{array}{c} \frac{W_{ij}{\left(MM^{T}+\mu ee^{T}\right)}_{ij}}{{\left(\left(C_{2}⊙G\right)MM^{T}+\mu \left(C_{2}⊙G\right)ee^{T}\right)}_{ij}},x_{j}\in N_{2}\left(x_{i}\right)\\ 0\;,x_{j}\notin N_{2}\left(x_{i}\right) \end{array}\right.$$

The final similarity matrix can be acquired by iterating until convergence or the maximum rounds.

#### Feature Extraction and Symbol Definition

Given a set of *l* lncRNAs, *l*_1_,…, *l*_*i*_,…, *l*_*l*_, and a set of *m* miRNAs, *m*_1_,…, *m*_*j*_,…, *m*_*m*_, whose interaction is represented by a matrix *Y* of *l* × *m*, if there exists an interaction between lncRNA *l*_*i*_ and miRNA *m*_*j*_, then *Y*_*ij*_ = 1; otherwise, *Y*_*ij*_ = 0.

Four features of lncRNAs and miRNAs are extracted (110 features in total). The frequency of *l* lncRNAs with *4^k^* long contiguous subsequences is calculated. Herein, we assumed *k* = 1, 2, 3 to obtain the lncRNA-related feature vector. The sequence similarity between pairs of *l* lncRNAs is calculated to yield the similarity matrix of *l* × *l*, which is denoted as *S_lncS*. In the same manner, the similarity matrix of *m* miRNAs is denoted as *S_miS* of *m* × *m*. The interaction profiles of lncRNAs and miRNAs are derived from the interaction matrix. For lncRNA *l*_*i*_, the interaction profile indicates whether it interacts with each miRNA, matching the *i*-th row of *Y*, i.e., *Y*(*i*,:). Similarly, for miRNA *m*_*j*_, it matches the *j*-th row of *Y*, i.e., *Y*(:, *j*). The similarity between two interaction profiles of *l* lncRNAs is calculated as the matrix *S_lncP* of *l* × *l*, whereas the similarity between two interaction profiles of *m* miRNAs is computed as the matrix *S_miP* of *m* × *m*.

#### Label Propagation Based on Improved Linear Neighborhood Similarity

Label propagation (Kato et al., 2009) assigns labels to previously unlabeled data points. During label assignment, the labels of labeled data points are propagated to unlabeled data points. The core idea of the label propagation algorithm is that similar nodes should have similar labels. It involves two stages, namely, calculating the similarity matrix and propagating the labels.

The edge from node *i* to node *j* represents the similarity of these nodes. All edge weights constitute a weight matrix, where the higher the similarity the larger the weight. Herein, ILNS is adopted to construct the similarity matrix and calculate the Cosine-distance neighbors and the Euclidean-distance neighbors of each node until convergence. Nearer neighbors of each data point are fixed to a certain proportion, and the weights of others are 0. The weight matrix is actually a sparse matrix. The labels are propagated through the edges between the nodes. The larger the weight of the edge, the more similar the nodes are to each other and the easier to propagate the labels (Zang and Zhang, 2012; Zhang et al., 2016). For *m* data points *x*_1_,…, *x*_*m*_, an *m* × *m* probability transfer matrix *P* is defined as


(14) $$P_{ij}=P\left(i\to j\right)=\frac{w_{ij}}{\sum_{k=1}^{m}w_{ik}}$$

where *P*_*ij*_ represents the transferring probability and *w*_*ij*_ is the weight. The propagation involves three steps. First, a unique label is allocated to each node, i.e., label one for node one and label *i* for node *i*, where the labels are different from each other. Second, for node *j*, all nodes are traversed to discover their neighboring nodes and obtain their labels to obtain the label with the most occurrences. If more than one label satisfies the largest number of occurrences, then one is randomly selected to replace the current label. Finally, if the label of node *j* no longer changes after this round of relabeling or the pre-set number of rounds is reached, then the iteration is stopped. Otherwise; step 2 is repeated.

#### Prediction Model Multi-Source Information-Based Linear Neighborhood Propagation

**FIGURE 2:**
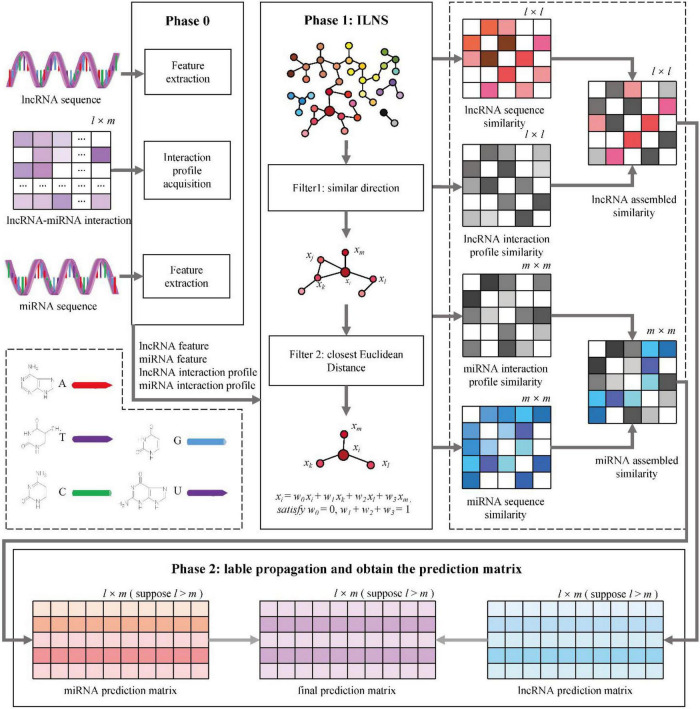
MILNP for predictions. Phase 0: Extraction of sequence features and interaction profiles. Phase 1: Calculation of sequence similarity and interaction profile similarity to generate integrated similarity. Phase 2: Label propagation using weighted sum to obtain the final prediction matrix.

The sequence and interaction profiles of lncRNAs and miRNAs are captured to develop our model. The workflow of MINLP is shown in Figure 2. The specific steps are as follows:

Step 1: the sequence feature similarity *S_lncS* and *S_miS* are calculated using the ILNS algorithm.

Step 2: the interaction profiles of all lncRNAs and all miRNAs are exported according to the interaction matrix of lncRNAs and miRNAs.

Step 3: the interaction profile similarity *S_lncP* and *S_miP* are calculated using the ILNS algorithm.

Step 4: *S_lncS* and *S_lncP* are combined to obtain lncRNA integrated similarity, and *S_miS* and *S_miP* are combined to obtain miRNA integrated similarity.

Step 5: for the two integration similarities, the linear neighborhood propagation method is used to generate the prediction of lncRNA and the prediction of miRNA.

Step 6: the weighted sum of the two prediction matrices is calculated, and the final interaction prediction matrix is determined.

## Experiments and Results

### Evaluation Criteria

The criteria for measuring prediction models are area under curve (AUC), area under precision–recall (AUPR), REC, SPE, and ACC. AUC is the area under the receiver operating curve (ROC) coordinated by true positive rate–false positive rate, which is suitable for observing model performance in the case of a balanced positive and negative sample size. The formulae of REC, SPE, and ACC are as follows.


(15) $$\left\{ \begin{array}{c} REC=\frac{TP}{TP+FN}\\ SPE=\frac{TN}{FP+TN}\\ ACC=\frac{TP+TN}{TP+FN+FP+TN} \end{array}\right.$$

The performance is evaluated *via* fivefold cross-validation. To achieve more accurate outcomes, each fivefold cross-validation is repeated for 20 rounds to ensure that a sufficient number of learnings are reached. *K*-fold cross-validation is frequently used to upgrade model performance, where data is divided into *K* equal parts, one of which acts as test data and the other acts as training data. A distinct test set is selected each time, and the rest serves as a training set. Finally, the results of *K* experiments are averaged.

### Parameter Settings

A total of four relevant parameters are obtained in this work.

In the computation of ILNS, Cosine distance-based neighbors (With the ratio of *K*_1_) and Euclidean distance-based neighbors (With the ratio of *K*_2_) are computed. *K*_1_ is set to {0.1, 0.2,…, 0.9}, whereas *K*_2_ is set to {0.1, 0.2,…, 1}, and their step size is 0.1. The purpose of this arrangement is to ensure that *K*_2_ considers all neighbors generated by *K*_1_, regardless of the size of *n*_1_. During label propagation, the parameter α is set as the probability of label absorption, i.e., for node *x*_*j*_ the probability of absorbing the label of its nearest neighbor node *x*_*i*_ is α. The value of α is set within the range {0.1, 0.2,…, 0.9}, and the step size is 0.1. After the lncRNA prediction matrix *SL* and the miRNA prediction matrix *SM* are figured out, β is the trade-off parameter, i.e., the final prediction matrix will be measured as β × *SL* + (1 – β) × *SM*. The value of β is within {0.0, 0.05,…, 1.0}, and the step size is 0.05.

The settings of the parameters are shown in Table 2. Subsequent experiments are conducted with the most optimal parameter combinations.

**TABLE 2:** Parameter setting.

Phase	Parameter	Range	Step length
Phase 1	*K*_1_	0.1 — 0.9	0.1
Phase 1	*K*_2_	0.1 — 1.0	0.1
Phase 2	α	0.1 — 0.9	0.1
Phase 2	β	0.0 — 1.0	0.05

### Results

#### Optimal Parameters

**FIGURE 3:**
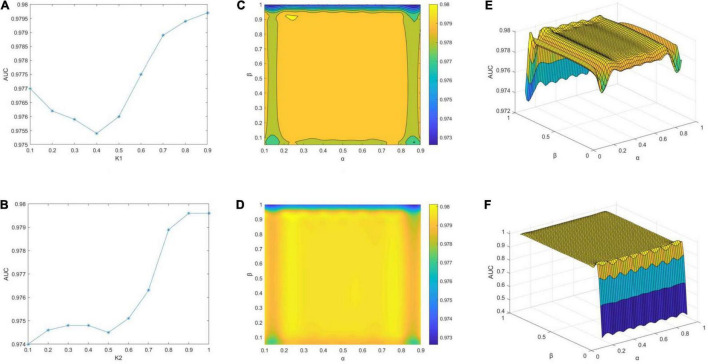
Impact of parameters on AUC scores of MILNP. **(A)** Effect of *K*_1_ when fixing *K*_2_, α and β. **(B)** Effect of *K*_2_ when fixing *K*_1_, α and β. **(C–F)** Effect of α and β when fixing *K*_1_ and *K*_2_.

The effects of the different parameters are visualized in Figure 3. First, α and β are fixed. Theoretically, the neighbors are set twice to find a more accurate batch faster. However, serious analysis reveals that a change in *K*_2_ is logical within a certain range of *K*_1_; otherwise, even when *K*_2_ is 100%, it will not be very helpful. Let *K*_2_ = 1.0, as *K*_1_ changes from 0.1 to 0.9, we find that *K*_2_ = 0.9 is the optimal value, as shown in Figure 3A. AUC initially decreases and then increases with *K*_1_ and reaches its lowest point at 0.4. The AUC values are always above 0.975. *K*_1_ = 0.9 is then fixed, and *K*_2_ is changed. As *K*_2_ becomes larger, AUC tends to increase globally and reaches the maximum at 0.9 (Figure 3B). The most pronounced increase is evident from 0.7 to 0.8, clearly demonstrating that 0.8 is a cut-off point. Finally, the impact of α and β is investigated. The contour and concentration plots in Figures 3C,D, respectively, demonstrate the variations in AUC with α and β. The gradient from blue to yellow is set to indicate the increase in AUC values. Herein, the scenario with β = 0 is dropped to detect subtle variations in the other cases owing to our prior observation that the AUC value is as low as 0.4 in the case of β = 0, thereby forming a cliff-like change from others. Both plots show that the best results are achieved at α of 0.2–0.8 and β of 0.15–0.9, which are extremely close to 0.98. The yellowest areas appear locally as a result of drawing tool error, but it does not affect the fact that the results are roughly consistent. Figures 3E,F present the grid plots of AUC with respect to variations in α and β, respectively. Figure 3E is the case with β = 0 removed from the corresponding plots in 3(C) and 3(D). Figure 3F includes all cases and validates the previous interpretation that the inclusion makes distinguishing the changes from the others difficult. The models of all ranges show that the best parameters are *K*_1_ = 0.9, *K*_2_ = 1.0, α = 0.7, and β = 0.35 when AUC at this point is 0.9797. This tells that the performance of our model is attributed to Cosine distance, determining a more accurate neighborhood which is preferable to applying Euclidean distance only.

#### Control Information Sources and Similarity Measurement Algorithm

To demonstrate the superiority of our model, we calculate a similarity network with a single information source and build linear neighborhood propagation models to compare with MILNP. The results are presented in Table 3. The optimal values obtained are set in bold typeface. We construct these models based on the optimal combination of parameters. Except for the difference in information sources, all the other processes are guaranteed to be the same. The model using only sequence similarity with ILNS as the core algorithm is named MILNP-I. The model using only interaction profile similarity with ILNS as the core algorithm is named MILNP-II. Overall, both of them are inferior to MILNP with integrated information. MILNP-II is generally very close to our model, indicating that the interaction information is a key contributor throughout the prediction. Thus, with specific optimization, MILNP-II could be credible for predicting isolated lncRNAs or miRNAs. The performance of MILNP-I is not as good as that of MILNP-II, but its AUC is barely satisfactory. We also tried another method for calculating similarity, LNS (Zhang et al., 2018b), to obtain new models, named the SLNPM-series, as shown in Table 3. With the information source controlled, a comparison of SLNPM-II and MILNP-II reveals that ILNS is more accurate. All these results clearly validate the superiority of MILNP in terms of information integration and similarity calculation.

**TABLE 3:** Performance of models with combinations of different algorithms and information.

Model	Algorithm	Information	AUC	REC	SPE	ACC	AUPR
SLNPM-I	LNS	Sequences similarity	0.8596	0.2883	0.9962	0.9932	0.1856
SLNPM-II	LNS	IP similarity	0.8756	0.5993	0.9990	0.9973	**0.5981**
SLNPM	LNS	Sequence similarity and IP similarity	0.9768	0.9613	0.9993	0.9993	0.5132
MILNP-I	ILNS	Sequence similarity	0.8561	0.4620	0.9902	0.9916	0.1249
MILNP-II	ILNS	IP similarity	**0.9804**	0.9600	0.9994	0.9994	0.5235
MILNP	ILNS	Sequence similarity and IP similarity	0.9797	**0.9629**	**0.9994**	**0.9994**	0.5297

#### Comparison With Existing Methods

As far as we know, very few studies have investigated lncRNA–miRNA interactions in plants. We select Pmlipred (Kang et al., 2020) and CIRNN (Zhang et al., 2020) as reference methods. Both methods predict plant lncRNA–miRNA interactions. PmliPred (Kang et al., 2020) builds a prediction model by using a machine learning approach combined with a deep learning approach, and the final prediction results are made from fuzzy decisions of the two components. We use the publicly available source code on GitHub to implement Pmlipred (Kang et al., 2020). CIRNN (Zhang et al., 2020) builds integrated deep learning models with both a CNN and an IndRNN, where the former is used to automatically extract gene sequence functional features, whereas the latter is utilized to obtain sequence feature representations and dependencies. We replicate it in the detailed description of CIRNN (Zhang et al., 2020). The results of the comparison are summarized in Table 4. The optimal values obtained are set in bold typeface. The performance of the two methods is clearly good, but not as good as that of our model. In particular, the AUC and ACC values have a relatively large gap. We notice that both methods stretch to deep learning. Thus, we implement similar models on their basis to measure their effectiveness. We construct a CNN–Gate Recurrent Unit (GRU) combinatorial model. Sequential features are extracted from the original data by CNN and compressed into a one-dimensional vector in the flattened layer to input into GRU. This process is well-suited for processing sequential information. We consider GRU instead of others because GRU has fewer parameters and reduces overfitting. We first use a three-layer CNN and a single-layer GRU mixed with RF to obtain CNNRF1. We then add another layer of CNN on top of that to obtain CNNRF2. Gladly, although our MILNP is simple and built by deriving mathematical formulas *via* a top–down approach and layer by layer, the results are satisfactory. For the baseline SLNSM (Zhang et al., 2018b) that is originally created for animal prediction, we run our dataset and observe that our method is slightly better. That is because we focus on improving the computational procedure for linear neighborhood similarity by adding the spatial direction restriction. The optimal parameter combination shows that such performance is attributed to Cosine distance, determining a more accurate neighborhood, which is preferable to the approach of the baseline.

**TABLE 4:** Performances of different methods.

Methods	AUC	REC	SPE	ACC	AUPR
Pmlipred	0.8386	0.9493	0.9087	0.9290	0.4304
CIRNN	-	0.9413	-	0.9604	-
CNNRF1	0.8562	0.9531	0.9083	0.9307	0.4321
CNNRF2	0.8284	0.9597	0.9047	0.9322	0.4340
SLNPM	0.9768	0.9613	0.9993	0.9993	0.5132
MILNP	**0.9797**	**0.9629**	**0.9994**	**0.9994**	**0.5297**

#### Case Study and Discussion

**FIGURE 4:**
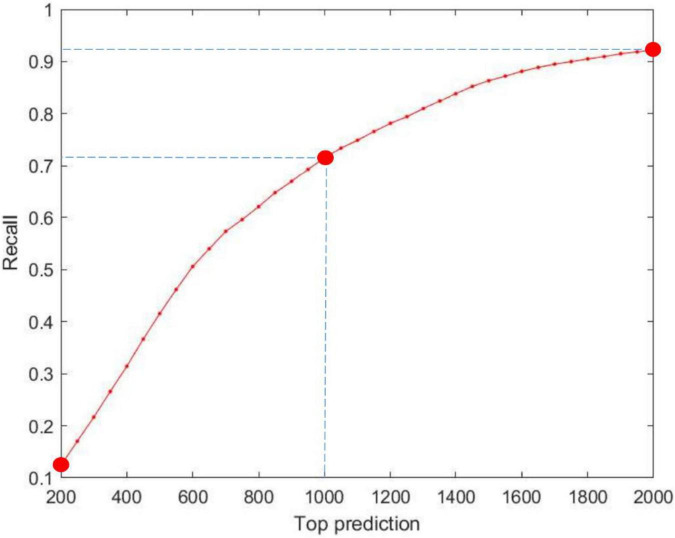
Performance of MILNP’s top-rank predictions, where the *X*-axis refers to the top 200 to top 2,000 predictions and the *Y*-axis refers to the recall generated by MILNP.

Top-rank prediction is an important way of visualizing the performance of the models. We examine the top-rank predictions from 200 to 2,000 and identify the percentage of interactions that are truly correct. As shown in Figure 4, an average of 186 positive interactions per prediction is reached in the top 200 predictions, whereas 1,380 real interactions are determined in the top 2,000 predictions. The results demonstrate the good performance of our model.

Furthermore, we predict the interactions of isolated lncRNAs and miRNAs with MILNP. For the isolated lncRNA or miRNA, only sequence-dependent information can be used. In separate cases gma-miR395a and lcl| Gmax_Glyma.18G279100.1 are taken as examples. We validate the prediction of the selected miRNA and lncRNA with respect to RNAhybrid2.1.2 (Rehmsmeier, 2004). All predictions are sorted in descending order of probability. For miRNA gma-miR395a, 4 of the top 10 are correctly predicted, as shown in Table 5, thereby confirming the predictive power of MILNP. However, the list reveals that the fifth and eighth detected lncRNAs in the prediction of miRNA “gma-miR395a” belong to *Medicage truncatula*, as evidenced by their nomenclature. The situation is worth contemplating. On the one hand, this indicates that the selected samples may affect the performance of MILNP. On the other hand, it inspires us to further explore cross-species linkages and assume that the remaining uncertified interactions are possible. Likewise, we make predictions for lncRNA lcl| Gmax_Glyma.18G279100.1. To our surprise, five of the results happen to be identified by the tool as having interactions, a result that is very encouraging. We conjecture that the remaining ones predicted by our model can be possible. As demonstrated by the results of the comparison of the two sets of predictions, the fact that sample selection has a great influence on the prediction results should not be ignored. The association between the selected sample and other samples also affects the results. For those that have a similarity with many samples, the prediction results may be more accurate. If a sample has little similarity with other samples, then predicting its potential interactions will be difficult.

**TABLE 5:** Top 10 predictions for miRNA “gma-miR395a” and lncRNA “lcl| Gmax_Glyma.18G279100.1” by MILNP.

NO	gma-miR395a	Confirmation	lcl| Gmax_Glyma.18G279100.1	Confirmation
	**lncRNAs**		**miRNAs**	
1	lcl| Gmax_Glyma.19G246900.1	NO	gma-miR319a	YES
2	lcl| Gmax_Glyma.15G199100.1	NO	gma-miR319h	YES
3	lcl| Gmax_Glyma.14G142000.1	YES	gma-miR319g	YES
4	lcl| Gmax_Glyma.08G153500.1	YES	gma-miR319i	NO
5	lcl| Mtruncatula_Medtr1g017330.1	NO	gma-miR319p	NO
6	lcl| Gmax_Glyma.16G164700.2	NO	gma-miR319c	YES
7	lcl| Gmax_Glyma.07G234600.1	YES	gma-miR319q	NO
8	lcl| Mtruncatula_Medtr8g099205.1	NO	gma-miR159a-3p	YES
9	lcl| Gmax_Glyma.12G192900.2	YES	gma-miR319f	NO
10	lcl| Gmax_Glyma.02G080100.1	NO	gma-miR5676	NO

The different results also prompt us to reconsider the results of previous experiments. We find that, although MILNP achieves good AUC and ACC, its PRE is relatively low, which may be attributed to the model itself and the distribution of the dataset. Some very similar samples may have confused the model and that induces it to arrive at a wrong judgment. Nevertheless, it could also be a new revelation that suggests these possible associations. This assumption warrants further biological laboratory validation.

## Conclusion

lncRNA–miRNA interactions are important because they influence various biological activity processes. Most studies on these interactions focused on animals. Although experimental results derived from studies of plants are not as easy to verify as those obtained from animals, current research is not merely a conjecture. Great improvements have been made by scientists after proposing bold assumptions and providing carefully evaluated proofs. Herein, we attempt to study plant interactions and propose a linear neighborhood propagation model based on combinatorial information. We validated it on datasets of three plants. We have obtained relatively good results. More importantly, we proposed a novel method for measuring similarity from mathematical fundamentals. We used the combined information of molecular sequences and interactions to construct a similarity network with a guarantee of being nearer in both spatial location and direction. We achieved the final prediction by label propagation. A series of experiments showed the outstanding performance of our model, demonstrating the superiority of the combinatorial information. We also attempted to predict isolated lncRNAs and miRNAs without any interaction yet and validated the predictions with existing tools. Our model possesses good generalization properties and can be used to discover new interaction relationships. Our multisource information-based linear neighborhood propagation method is a novel and unique method for predicting plant lncRNA–miRNA interactions. However, the entire study requires a large time investment of about 3 months. Hence, in a follow-up study, we will tune the parameters to make the model more efficient. We will also consider deep learning methods on this basis and combine the results that we may obtain.

## Data Availability Statement

The original contributions presented in the study are included in the article/supplementary material, further inquiries can be directed to the corresponding author/s.

## Author Contributions

MG wrote the first draft of the manuscript. XF wrote sections of the manuscript. All authors contributed to manuscript revision, read, and approved the submitted version.

## Conflict of Interest

The authors declare that the research was conducted in the absence of any commercial or financial relationships that could be construed as a potential conflict of interest.

## Publisher’s Note

All claims expressed in this article are solely those of the authors and do not necessarily represent those of their affiliated organizations, or those of the publisher, the editors and the reviewers. Any product that may be evaluated in this article, or claim that may be made by its manufacturer, is not guaranteed or endorsed by the publisher.
